# Effectiveness of a Health Awareness Program for Early Detection of Breast Cancer Among Female Patient Attendants in a Selected Hospital in North-East India: A Preexperimental Pretest–Posttest Study

**DOI:** 10.7759/cureus.103446

**Published:** 2026-02-11

**Authors:** Mayengbam B Devi, Jagannath D Sharma, Monoj K Baruah, Krishangi Kashyap, Kaberi Saikia, Putul Mahanta

**Affiliations:** 1 Nursing, Rahman Institute of Nursing and Paramedical Sciences, Guwahati, IND; 2 Oncopathology, State Cancer Institute, Guwahati, IND; 3 Obstetrics and Gynecology, PA Sangma International Medical College and Hospital, Guwahati, IND; 4 Oncology Nursing, Nalbari Medical College and Hospital, Nalbari, IND; 5 Nursing, Assam Royal Global University, Guwahati, IND; 6 Forensic Medicine and Toxicology, Nalbari Medical College, Nalbari, IND

**Keywords:** breast cancer, breast self-examination, mammography, screening test, tertiary care center

## Abstract

Introduction

Breast cancer (BC) is the most frequent malignancy among women. Early diagnosis of BC can be achieved through breast self-examination (BSE) and breast cancer screening tests (BCST). BC prevention is most effective when people are aware of the disease. The present study aims to assess the effectiveness of a health awareness program for early BC detection among the female attendants of admitted patients at a tertiary care center.

Methods

The study adopted a preexperimental one-group pretest-posttest design and included 450 female attendants of admitted BC patients at the Dr. Bhubaneswar Borooah Cancer Institute, Guwahati, India (Tata Memorial Centre). The participants were selected using non-probability convenience sampling. The data collection tools included the demographic proforma; the knowledge questionnaire for BC, BSE, and BCST; the skill checklist for BSE; and the practice questionnaire for BC and BCST. On the first day, a pretest was administered. Following the pretest, a health awareness program focused on early detection of BC was delivered. The health awareness program included lectures and discussions covering the anatomy of the breast, risk factors, signs and symptoms, diagnosis, treatment, and prevention of BC; the meaning of BSE; when to perform BSE; changes to look for during BSE; what to do if a lump is discovered during BSE; and a demonstration of BSE using a breast model. The health awareness program lasted 45 minutes. The posttest was completed on the eighth day using the identical instruments as the pretest, except for the demographic proforma. Descriptive and inferential statistics were used to analyze the data. The difference between mean scores at pretest and posttest was tested using a paired t-test or Wilcoxon signed-rank test, while the difference between proportions was compared using a z-test for two proportions or McNemar's test, as per the normality of the data.

Results

Of the 450 participants, 339 (75.3%) were in the 20-30-year age group, and 315 (70.0%) were from rural areas. Most of the participants were literate, with 172 (38.2%) holding a high school diploma, and the majority belonged to low-income families and were unemployed (n = 283, 62.9%). Most participants (278; 61.8%) had heard of BC, and they primarily learned about it from the media (n = 251; 55.8%). The majority (393, 87.3%) had no family history of BC. At the pretest, 232 (51.6%) participants had average knowledge of BC, BSE, and BCST. Furthermore, 417 (92.7%) had poor BSE skills. After the health awareness program, the majority (319, 70.9%) of participants demonstrated excellent knowledge. At posttest, 324 (72.0%) demonstrated excellent BSE skills. A statistically significant difference was observed between the pretest and posttest knowledge scores (Z = -18.10, p < 0.01). The mean BSE skill scores of participants increased significantly from 3.32 at pretest to 19.08 at posttest. Only 11.6% (n = 52) of participants had done BSE at pretest, which increased to 17.6% (n = 74) at posttest. Only 23 (5.1%) participants reported regular BSE practice at posttest. None of the female participants had ever undergone mammography.

Conclusions

The health awareness training on early identification of BC improved participants' understanding of BC, BSE, and screening tests, as well as their ability to perform BSE. The prevalence of BSE and BCST was extremely low and sporadic among the participating females.

## Introduction

Breast cancer (BC) is the most frequent cancer diagnosed in women, accounting for more than one in every 10 new cancer diagnoses each year, and is the second most common cause of cancer death among women globally. In 2022, an anticipated 2.3 million new BC cases and 666,000 BC-related fatalities were reported worldwide, accounting for 23.8% and 15.4% of all cancer diagnoses and deaths in women, respectively [1]. BC affects women of all ages after puberty; prevalence increases with age. In 2020, BC accounted for 10% of cancer-related deaths and 13.5% of new cancer cases among females in India, making it the country's largest cause of cancer incidence and mortality [2]. Among all population-based cancer registries in northeastern India, Aizawl district in Mizoram had the highest BC incidence, whereas Kamrup urban district in Assam ranked third, with an age-adjusted incidence rate of 27.1 [3]. Although it is one of the leading cancer sites among females in Assam, the BC screening rate is only 0.2% [4].

The WHO announced the Global Breast Cancer Initiative (GBCI) in 2021 with the aim of lowering population-level, age-standardized BC death rates by 2.5% each year through 2040 by encouraging multisectoral partnerships, sustained capacity building, and the implementation of monitoring systems for decision-making [5]. Early detection, timely diagnosis, and comprehensive BC management are the three pillars of health promotion for achieving the goal. Health education is crucial for raising women's knowledge of the signs and symptoms of BC and for helping them and their families understand the need for early detection and treatment, so that more women visit medical practitioners when BC is first suspected and any cancer present is advanced. This is conceivable even without mammography screening, which many countries cannot currently implement [6].

BSE is a straightforward, inexpensive, non-invasive procedure that does not require specialized supplies or equipment and is a useful method for diagnosing BC. BSE is a practical method for early BC screening in underdeveloped countries. Globally, there is a significant knowledge-application gap between what is known and how BSE is actually practiced [7]. The low uptake of BSE has been attributed to several sociodemographic factors, myths, cultural beliefs, and limited access to healthcare facilities. A mammogram is considered the gold standard screening, which can often detect breast changes that could be cancer years before physical symptoms develop [8].

Women's health behaviors, including BSE, mammography, and Pap smear exams, were found to be positively impacted by education about the early detection of breast and cervical cancer [6]. Major barriers to cancer screening include lack of knowledge, the cost of screening services, and distance to the screening facility. Screening may be hampered by cultural factors, such as a lack of family or spouse support, and embarrassment and psychosocial factors, such as fear of the screening procedure and fear of being diagnosed with cancer. To boost screening adherence, women should be educated about the causes and risk factors of cancer through evidence-based interventions [9]. It is necessary to raise women's awareness of the importance of early BC detection through screening. The information they learn about BC and BCST along with the BSE skills they acquire through awareness interventions will enable the women to recognize the early indicators of BC and seek medical attention at an early stage. Therefore, the present study aims to assess the effectiveness of a health awareness program in increasing knowledge of BC, BSE, and BCST and to examine its effects on BSE skills and screening practices among female patient attendants of admitted patients.

The primary objective of the present study was to assess the effectiveness of a health awareness program in improving knowledge of BC, BSE, and BCST among female patient attendants. The secondary objectives were (1) to evaluate the program's effect on BSE skills using a structured checklist and (2) to assess changes in BSE and screening practices eight days after the intervention.

## Materials and methods

In the present study, a quantitative research approach with a pre-experimental pretest-posttest design was employed. The study was conducted among female patient attendants of patients admitted to various wards of BBCI, Guwahati, India. A non-probability convenience sampling technique was used to select the participants. Ethical clearance was obtained from the BBCI Medical Ethics Committee under reference no. ECR/1040/Inst/AS/2018, dated October 19, 2020. Permission was obtained from the director of BBCI to collect data. Informed consent for voluntary participation was obtained from all the participants. The study was conducted between January 2021 and February 2024.

Sample size

To estimate the sample size, the standard formula for estimating a single proportion was used:

N = Z^2^α × p × q/d^2^

where Zα = 1.96 for 95% confidence level, p = anticipated prevalence, q = 1-p, and d = relative error.

Assuming that at least 20% of the general population is aware of BC, with a 4% margin of error to allow for a 20% relative error in the population prevalence and a 10% non-response rate, the sample size was estimated at 423 with a 95% confidence level. Finally, the study's total sample was set at 450 to partially compensate for the variability introduced by convenience sampling.

Inclusion and exclusion criteria

The study focused on female patient attendants of admitted patients aged 20-50 who can understand, read, and write Assamese and/or English. The study did not include female patient attendants with BC or health professionals. Those who were not willing to participate in the study were also excluded.

Data collection tools

Four data collection tools were used. The first tool was the demographic proforma, which consisted of seven items: age, education, occupation, monthly family income, awareness of BSE, source of information, and family history of BC. The second tool was a knowledge questionnaire on BC, BSE, and BCST, consisting of 20 multiple-choice questions. Each correct response scored 1, and each incorrect response scored 0, with a total score of 20. The knowledge score, ranging from 0 to 5, is graded as poor knowledge, 6-10 as average knowledge, 11-15 as good knowledge, and 16-20 as excellent knowledge. The third tool was the BSE skill checklist. Each correctly performed step scored 1, and each incorrectly performed step scored 0. The maximum score was 22, and the minimum score was 0. The skill score of 18-22 (above 80%) is categorized as excellent skill, 11-17 (50% to 80%) as good skill, and 0-10 (below 50%) as poor skill in BSE. The fourth tool was the BSE and BCST practice questionnaire.

Content validity of the study tools was assessed by seven experts from the medical, surgical, and nursing fields, who evaluated the items for clarity, relevance, meaningfulness, and content. Necessary modifications and reorganization of the items were implemented in accordance with the experts' recommendations. The reliability of the tools was assessed among 20 female patient attendants caring for admitted patients. The reliability of the knowledge questionnaire on BC, BSE, and BCST was assessed using the split-half method and the Pearson product-moment correlation coefficient. The tool's reliability coefficient was 0.8. The reliability of the skill checklist on BSE was assessed using the inter-rater method and quantified using the Pearson correlation coefficient. The reliability coefficient was 0.9. The reliability of the BSE and BCST practice questionnaire was assessed using the test-retest method and calculated using Karl Pearson's correlation coefficient. The reliability coefficient was 0.8. The feasibility of the study was assessed through a pilot study involving 100 female patient attendants. The time each female patient attendant took to complete the data collection tools was about six to eight minutes. The skill observation took around 8-10 minutes.

The health awareness program on early detection of BC

The awareness program was given individually or in groups in different wards of BBCI. The health awareness program lasted 45 minutes. The health awareness program was delivered by one trained nurse using audiovisual aids, including a PowerPoint presentation and a demonstration of BSE using a dummy breast model.

The awareness program included lectures and discussions on the following topics: (a) brief anatomy of the breast, including details about lobes, lobules, ducts, nipple, areola, connective tissue and ligaments, blood vessels, lymph vessels, and lymph nodes, etc.; (b) risk factors of BC, including details about age, gender, genetic risk factors, anatomical factors, alcohol consumption and other dietary factors, early menstruation, late menopause, late childbirth or never being pregnant, hormonal therapy, and previous BC; (c) signs and symptoms of most common BCs including a breast lump or tissue thickening that feels different than surrounding tissue and has developed recently; breast pain; red, pitted skin over entire breast; swelling in all or part of breast; a nipple discharge other than breast milk; bloody discharge from the nipple; peeling, scaling, or flaking of skin on nipple or breast; a sudden, unexplained change in the shape or size of breast; inverted nipple; changes to the appearance of the skin on breasts; a lump or swelling under arm, etc.; (d) different methods of diagnosis of BC, including detailed discussion on mammograms, ultrasounds, MRIs, and breast biopsies; and (e) the treatment options for BC, including details about the various types of surgeries and biopsy procedures, radiation therapy, chemotherapy and hormone therapy, and different prevention measures. PowerPoint slides were used to explain the meaning of BSE and when to perform it; changes to look for during BSE, such as the development of a lump; discharge other than breast milk; swelling of the breast; skin irritation or dimpling; and nipple abnormalities (such as pain, redness, scaliness, or turning inward); steps for performing BSE; and how to respond if a lump is discovered during BSE. Finally, a BSE demonstration with a breast model was given.

A pretest was conducted on the first day. After the pretest, the health awareness program on early detection of BC was given. The posttest was conducted on the eighth day using the same tools used in the pretest, except for the demographic proforma. The BSE skill assessment was performed on a breast model with one normal breast and one with a lump, using inspection and palpation. Two trained nurses observed the BSE skills, and the observation checklist was filled out. Participants were asked to submit responses to all study questions to prevent data loss due to missing or incomplete responses.

Statistical analysis

The data were analyzed using the Statistical Package for Social Sciences (SPSS), version 21 (IBM Corp., Armonk, NY). The mean and standard deviation (SD) were used to present the continuous variables. Categorical variables were presented as percentages and frequencies. The normality of data was tested using Kolmogorov-Smirnov test and the Shapiro-Wilk test. The pre- and posttest scores were compared using a paired t-test or a Wilcoxon signed-rank test, depending on the normality of the data. The z-test or the nonparametric McNemar test was used to compare two proportions. A p-value below 0.05 was deemed statistically significant.

Furthermore, the gains in knowledge and skill levels due to the health awareness program were presented as actual, possible, and modified gain scores. As per the standard formulas, the actual gain score was represented as the difference between the maximum score and the posttest mean score. The possible gain score was the difference between the maximum score and the pretest mean score. The modified gain score was the ratio of the possible gain score to the actual gain score.

## Results

Most study participants were from rural areas (n = 315; 70.0%) and were aged 20-30 years (n = 339; 75.3%). The participants were mostly literate, with 172 (38.2%) holding a high school certificate and another 130 (28.9%) studying up to the graduation level. The participants were mostly unemployed (n = 283, 62.9%), and the majority were from low-income families (n = 120, 26.7%). Of the 450 female attendants, 278 (61.8%) have heard of BC. Media (newspaper, radio, television, etc.) was the most reported source of information (n = 251, 55.8%). There was no family history of BC among 393 (87.3%) female patient attendants, as shown in Table 1.

**Table 1:** Sociodemographic profile of the study participants The data have been presented as frequencies (n) and percentages (%); total sample size, N = 450.

Variables	Categories	Frequency (%)
Residence	Urban	135 (30.0%)
	Rural	315 (70.0%)
Age in years	20-30	339 (75.3%)
	31-40	98 (21.8%)
	41–50	13 (2.9%)
Education	Illiterate	39 (8.7%)
	Primary school certificate	29 (6.4%)
	Middle school certificate	32 (7.1%)
	High school certificate	172 (38.2%)
	Intermediate or diploma	8 (1.8%)
	Graduate	130 (28.9%)
	Professionals or honors	40 (8.9%)
Occupation	Unemployed	283 (62.9%)
	Elementary occupation	36 (8.0%)
	Plant and machine operators and assemblers	2 (0.4%)
	Skilled workers and shop & market sales workers	21 (4.7%)
	Clerks	10 (2.2%)
	Professionals	96 (21.3)
	Legislators, senior officials and managers	2 (0.4%)
Monthly family income in rupees	≤6323	120 (26.7%)
	6327-18,949	98 (21.8%)
	18,953-31,589	67 (14.9%)
	31,591-47,262	44 (9.8%)
	47,266-63,178	30 (6.7%)
	63,182-126,356	36 (8.0%)
	>126,360	55 (12.2%)
Family history of breast cancer	Yes	57 (12.7%)
	No	393 (87.3%)
Heard of breast cancer	Yes	278 (61.8%)
	No	172 (38.2%)
Source of information	Media (newspaper, radio, television, etc.)	251 (55.8%)
	Friends	49 (10.9%)
	Healthcare provider (specify)	70 (15.6%)
	Family members	14 (3.1%)
	Others	66 (14.7%)

As shown in Table 2, the majority (232; 51.6%) of participants had average pretest knowledge. Furthermore, 92.7% (n = 417) of participants had poor BSE skills in the pretest. At the posttest, 70.9% of participants (n = 319) demonstrated excellent knowledge, indicating the effectiveness of the health awareness program. Similarly, the BSE skill level was excellent among 72.0% (n = 324) of participants in the posttest.

**Table 2:** Knowledge and skill level of participants before and after the health awareness program The data have been presented as frequencies (n) and percentages (%); total sample size, N = 450. BC: Breast cancer; BSE: Breast self-examination; BCST: Breast cancer screening tests.

Variables	Score categories	Pretest	Posttest
		Frequency (%)	Frequency (%)
Knowledge level on BC, BSE, and BCST	Excellent	21 (4.7%)	319 (70.9%)
	Good	145 (32.2%)	116 (25.8%)
	Average	232 (51.6%)	15 (3.3%)
	Poor	52 (11.6%)	0 (0.0%)
Skill level of BSE	Excellent	9 (2.0%)	324 (72.0%)
	Good	24 (5.3%)	95 (21.1%)
	Poor	417 (92.7%)	31 (6.9%)

As the data did not follow normality, the pre- and posttest scores were compared using the Wilcoxon signed-rank test. The mean knowledge score significantly increased from 9.42 at the pretest to 17.04 at the posttest (Z-value = -18.10, p-value < 0.01). Additionally, the health awareness program significantly improved participants' average BSE skills, increasing from 3.32 at the pretest to 19.08 at the posttest (Z-value = -18.25, p < 0.01), as shown in Table 3.

**Table 3:** Effectiveness of health awareness program in terms of knowledge, skill, and practice level of the participants; N = 450 p < 0.05 was considered statistically significant for the Wilcoxon signed-rank test. Data have been presented as mean ± standard deviation (SD). BC: Breast cancer; BSE: Breast self-examination; BCST: Breast cancer screening tests.

Variables	Mean score		Paired difference		Z-value	p-value
	Pretest	Posttest	Mean	SD		
Knowledge level on BC, BSE, and BCST	9.42 (±3.26)	17.04 (±2.68)	7.62	4.16	-18.10	<0.01
Skill level of BSE	3.32 (±4.04)	19.08 (±4.07)	15.76	5.73	-18.25	<0.01

The mean knowledge score percentage increased from 47.1% in the pretest to 85.2% in the posttest, with a modified gain score of 0.72, indicating a positive change in knowledge level after the health awareness program. Similarly, a marked improvement in BSE skill (modified gain score = 0.84 relative to baseline) was observed among participants following the health awareness program (Table 4).

**Table 4:** Gain in knowledge and skill scores of the participants after the health awareness program on early detection of breast cancer; N = 450 The data are presented as mean score and mean percentage (%). BC: Breast cancer; BSE: Breast self-examination; BCST: Breast cancer screening tests.

Variables	Maximum score	Pretest mean score (%)	Posttest mean score (%)	Actual gain score	Possible gain score	Modified gain score
Knowledge level on BC, BSE, and BCST	20	9.42 (47.1%)	17.04 (85.2%)	7.62	10.58	0.72
Skill level of BSE	22	3.32 (15.1%)	19.08 (86.7%)	15.76	18.68	0.84

The practice of BSE and BCST was low among the participants. Only 52 (11.6%) participants reported in the pretest that they had ever performed BSE. The frequency of participants with positive responses significantly increased in the posttest (n = 79, 17.6%). Additionally, the number of participants conducting regular monthly BSEs or on specific dates increased significantly after the awareness program (p-value < 0.01). None of the participants has ever undergone mammography in the present study (Table 5).

**Table 5:** Practice of BSE and BCST among the participants before and after the health awareness program The data has been represented as frequency of positive responses (n) and percentage (% total sample, N = 450). A p-value of less than 0.05 was considered statistically significant for the McNemar's test (using binomial distribution) for comparing two proportions. BC: Breast cancer; BSE: Breast self-examination; BCST: Breast cancer screening tests.

Practice of BSE and BCST	Response	Pretest (n = 450)	Posttest (n = 450)	p-value
		Frequency (%)	Frequency (%)	
Have you done BSE anytime?	Yes	52 (11.6%)	79 (17.6%)	<0.01
Did you do it regularly, once a month?	Yes	6 (1.3%)	23 (5.1%)	<0.01
Do you perform BSE a few days after your menstrual period, or do you choose a specific date each month if your periods are irregular or if you are in menopause?	Yes	17 (3.8%)	40 (8.9%)	<0.01
Have you ever had a mammography?	Yes	0 (0.0%)	0 (0.0%)	-

## Discussion

According to the WHO, BSE is a crucial part of an early diagnostic program and enables breast health awareness. BSE needs to be promoted, as the majority of breast tumors are self-detected. As part of their regular self-care routines, the BSE encourages women to take charge of their health [10]. In recent times, the medical community has moved away from this practice as studies show that BSE does not improve survival and can lead to unnecessary biopsies. In India, BSE is an essential technique for early diagnosis of BC, particularly in low-income environments. It helps women become more comfortable with their breasts and notice abnormal changes earlier. BSE increases early detection and is especially important in resource-constrained settings when combined with routine mammography and clinical tests. Frequent BSE can help identify cancer early, which is critical for improving survival rates. Although the effectiveness of BSE in reducing BC mortality in India remains uncertain, integrating BSE training into community health programs and combining it with clinical breast examination by qualified physicians may enhance early identification [11].

Of the 450 female patient attendants, 315 (70.0%) were from rural areas, indicating a predominance of the disease in the study region's rural areas. In contrast to the present findings, other studies have reported that most BC patients are from urban areas [12,13]. Most participants were in the 20-30-year age group (n = 339, 75.3%), consistent with previous research [14-17]. A few studies, however, reported a higher number of participants at a later reproductive age [18,19]. The participants were mostly literate, which makes them comparable to participants in previous studies [14-16,19]. Past studies reported that female participants were predominantly homemakers or unemployed, findings that align with our observations [15,16,20]. Almost 62.0% (n = 278) of female attendants in the current study have heard of BC. Other researchers also reported similar findings [14,15]. A majority (251, 55.8%) of the female attendants obtained information from the media. Mass media as a primary source of information on BC has previously been reported [15,16]. In the present study, no family history of BC was reported among the majority (393, 87.3%) of female patient attendants, consistent with other studies [15,20].

Most respondents (n = 232, 51.6%) had only average knowledge of BC, BSE, and BCST at the pretest. According to earlier Indian research, female participants, especially those from rural areas, frequently lack knowledge about the signs, symptoms, and risk factors of BC [19,21]. As reported in a previous study, 85.1% (n = 354) of rural female participants were unaware of BSE [21]. In contrast, some studies from African countries reported a higher proportion of females with adequate knowledge on BC and BSE [22-24]. A study from Bangladesh reported lack of knowledge as the key barrier to BSE practice among females [25]. A significant correlation was identified between exposure to health education and cancer screening [19]. Almost 71.0% (n = 319) of the participants in the present study showed an excellent level of knowledge in the posttest. There was a significant difference (Z = -18.10, p < 0.01) between the pretest and posttest knowledge scores on BC, BSE, and screening tests, indicating the effectiveness of the health awareness program. Consistent with our findings, another recent study reported a notable improvement in women's knowledge following the implementation of a health education program, underscoring the importance of health education strategies in increasing knowledge of lifestyle diseases, including BC [26]. Like our findings, another study from South India reported a 71.8% increase in knowledge of BSE and breast health following a health awareness intervention [27].

Regarding BSE skill, 417 (92.7%) participants had a poor pretest skill level, consistent with the findings of Dadzi et al. [24]. However, in the posttest, 324 (72.0%) participants demonstrated an excellent level of BSE skill. The mean skill score significantly increased from 3.32 at pretest to 19.08 at posttest. Consistent with our findings, a recent study reported substantial improvements in BSE skill after two months of training, with notable enhancements in image identification, BSE steps, and lump detection in breast models among women [28].

During the pretest (n = 398, 88.4%), most participants reported never having performed BSE, consistent with findings from other studies [14,20-24,29]. At the posttest, the proportion of participants practicing BSE significantly increased to 17.6% (n = 79), up from 11.6% (n = 52) at the pretest. In both the pretest and posttest, a tiny proportion of females performed regular BSE. Although the percentage of participants practicing BSE after the awareness intervention differed statistically significantly from the pretest value, the difference was minuscule. In contrast to our findings, another study found a significant percentage shift in BSE practices between the pretest and posttest (21.3% vs. 33.8%; p < 0.001) [25].

None of the female attendants reported undergoing mammography ever in both the pretest and posttest. A similar study [19] reported low BC screening rates among female participants. Consistent with our findings, a recent study conducted in Saudi Arabia found that although most participants were aware of self-breast examination and mammography, utilization rates were suboptimal, with fear and embarrassment acting as barriers to mammography screening uptake [13]. Psychological barriers influenced by social convictions and ignorance, such as fear, worry, embarrassment, shyness, and neglect, were documented to hinder cancer screening among women [18]. A recent study conducted among urban Indian women noted differences in awareness of cancer screening, even though there was a high level of awareness about BC. Most women reported that the BC screening was favorable and important for early detection. The study also highlighted that barriers, such as a shortage of female physicians, financial constraints, shyness, lack of awareness of symptoms, and knowledge gaps, should be addressed to encourage women's participation in BC screening [30].

In the present study, marked improvements in knowledge (modified gain score: 0.72) and BSE skills (modified gain score: 0.84) were observed among participants following the health awareness program. However, we need to address the participants' infrequent use of BSE and other screening methods. Health intervention initiatives, along with strategic awareness campaigns that promote the importance of BSE and early BC screening among vulnerable communities, may help facilitate early BC diagnosis in the research location.

Limitations

The present study adopted a pre-experimental, pretest-posttest research design. Hence, there were no control groups to compare the true effects of the health awareness program. Additionally, the present study was single-center and hospital-based, which limits the generalizability of the findings. The small absolute improvements in actual BSE practice and the lack of change in mammography uptake, despite substantial knowledge and skill gains, may be attributable to the study's short follow-up period. A short follow-up period limits the study's ability to assess long-term retention of knowledge or sustained behavior change. However, as the current study comprised female attendants of hospitalized patients, a longer follow-up of participants beyond the patients' hospital stay was not possible. Along with the above limitations, the pre-post test research design of the study makes it vulnerable to unintended biases, as participants' responses might be affected by the Hawthorne effect and social desirability effects.

Multicenter studies can be replicated with larger sample sizes and longer follow-up periods to generalize the findings better. Also, future studies can focus on identifying barriers to practicing self-examination, screening, and preventative measures for BC among females in the study region.

## Conclusions

The study demonstrated that the health awareness program on early detection of BC was successful in increasing knowledge of BC, BSE, and screening tests and in enhancing BSE skills among participants. We observed that the participating women practiced BSE and BCST at very low levels and in an irregular manner. Although the health intervention program significantly improved knowledge and skills, the research population's poor BSE practice habits remain a concern. Health intervention programs, along with strategic awareness initiatives on the importance of BSE and early BC screening among vulnerable populations, might help detect BC earlier in the study region.

## Appendices

The demographic proforma is shown in Table 6.

**Table 6:** Demographic proforma

Instruction: Kindly read the following statements carefully, select the best alternative from the given options, and tick (√) accordingly.			
Participant No.			
S. No.	Demographic details	Categories	Response
1	Residence	Urban	
		Rural	
2	Age	<20 years	
		>20 to <30 years	
		>30 to <40 years	
		>40 to 50 years	
3	Education	Illiterate	
		Primary school certificate	
		Middle school certificate	
		High school certificate	
		Intermediate or diploma	
		Graduate	
		Professionals or honors	
4	Occupation	Unemployed	
		Elementary education	
		Plant & machine operators and assemblers	
		Craft and related trade workers	
		Skilled agricultural and fishery workers	
		Skilled workers and shop & market sales workers	
		Clerks	
		Technicians and associate professionals	
		Professionals	
		Legislators and senior officials & managers	
5	Monthly family income in rupees	≤6323	
		6327-18,949	
		18,953-31,589	
		31,591-47,262	
		47,266-63,178	
		63,182-126,356	
		>126,360	
6	Have you heard of breast self-examination?	Yes	
		No	
7	If yes, the source of information	Media (newspaper, radio, television, etc.)	
		Friends	
		Healthcare provider	
		Family members	
		Others	
8	Any family history of breast cancer	Yes	
		No	

The knowledge questionnaire on BC, BSE, and BCST is shown in Table 7.

**Table 7:** Knowledge questionnaire on breast cancer, breast self-examination, and breast cancer screening test

Instruction: Kindly read the following statements carefully, select the best alternative from the given options, and tick (√) accordingly.			
Participant No.			
S. No.	Knowledge questions	Categories	Response
1	Breast self-examination is a method to detect	Breast cancer	
		Bladder cancer	
		Bone cancer	
2	The best time to perform breast self-examination is	Before the menstrual period starts	
		During menstruation	
		Just after the menstrual period	
3	How frequently should you perform a breast examination?	Once a week	
		Once a month	
		Once a year	
4	During breast self-examination, the presence of a lump can be identified by	Standing in front of a mirror and observing the breast	
		Palpating the breast using fingers	
		Directly observing the breast	
5	If menses are irregular/menopause, the best way to select a day for breast self-examination is to select	Any specific date of a month	
		A few days after menses	
6	Which part hand is used to conduct the breast self-examination successfully?	Whole hand	
		Three fingers	
		Both hand	
7	When examining the right breast, which hand is best for feeling for changes in the tissue?	Right hand	
		Left hand	
		Both hands	
8	During nipple examination in breast self-examination, which of the following is to be assessed?	Discharge from nipple	
		Retracted nipple	
		Both	
9	While palpating the breast with fingers, which of the following techniques can be used?	Circular way to cover the whole breast	
		Vertical technique to cover the whole breast	
		Both techniques	
10	A woman should examine her breasts while in the shower because	She might miss lumps	
		More lumps are visible when the breasts are wet	
11	If you find the following during a breast self-examination, you should consult a doctor.	Dimpling on the breast	
		Scaling of skin	
		Any one of the above	
12	The presence of breast lumps should be assessed in	Breast only	
		The breast and the area between the breasts	
		Breast, the area between the breast and the underarm	
13	What other symptoms should women look for while checking their breasts for lumps?	Tenderness	
		Mobility	
		Both	
14	Which of the following is true regarding age and breast cancer?	Risk is greater under the age of 35	
		Risk increases with age after 35	
		Age is not related to risk	
15	In which of the following groups would women be at greatest risk for developing breast cancer?	Women who have a family history	
		Women who smoke cigarettes	
		Women who breastfeed	
16	If a lump is felt during breast self-examination, a woman should?	Consult doctor	
		Leave it and see if it reduces on its own	
		Apply local medicine	
17	Firm pressure is used during palpation to feel the tissue	Tissue closest to the skin	
		Tissue a little deeper	
		Tissue closest to the chest and ribs	
18	Mammography helps to detect cancer by	Using low-energy X-rays to examine the human breast	
		Extracting some tissue from the best	
		Injecting medicine into the breast	
19	At what age mammography be done?	Above 20 years	
		Above 30 years	
		Above 40 years	
20	How much time does it take to perform a mammography?	Approximately 5 minutes	
		Approximately 15 minutes	
		Approximately 30 minutes	

The skill checklist for BSE is shown in Table 8.

**Table 8:** Skill checklist for breast self-examination (BSE)

S. No.	Steps	Yes	No	Remarks
1	Stand in front of a mirror.			
2	Check both breasts for anything unusual.			
3	Look for discharge from the nipple, puckering, dimpling, or skin scaling.			
4	Watch closely in the mirror, clasp the hands behind the head, and press the hands forward.			
5	Note any changes in the contour of the breasts.			
6	Press the hand firmly on the hips and bows slightly toward the mirror.			
7	Note any changes in the contour of the breasts.			
8	Squeeze the nipple for any discharge.			
Examination of the right breast				
9	Lie flat on back, with right hand over head and a pillow or folded towel under the shoulder.			
10	Use the pads of the three fingers of the left hand to feel the right breast, applying firm, even pressure.			
11	Beginning at the outer edge, press the flat part of the fingers in small circles/vertically/wedge.			
12	Move fingers in circles/vertically/wedge around the breast to cover the whole breast.			
13	Gradually work toward the nipple.			
14	Palpate the underarm.			
15	Feel for unusual lumps or masses under the skin.			
Examination of the left breast				
16	Lie flat on back, with left hand over head and a pillow or folded towel under the shoulder.			
17	Use the pads of the three fingers of the right hand to feel the left breast, applying firm, even pressure.			
18	Beginning at the outer edge, press the flat part of the fingers in small circles/Vertically/wedge.			
19	Move fingers in circles/vertically/wedge around the breast to cover the whole breast.			
20	Gradually work toward the nipple.			
21	Palpate the underarm.			
22	Feel for unusual lumps or masses under the skin. (Reports feeling of two lumps/masses as per the model).			

The practice questionnaire for BSE and BCST is narrated in Table 9.

**Table 9:** Practice questionnaire for breast self-examination and breast cancer screening test

S. No.	Practice questions	Categories	Response
1	Have you done a breast self-examination anytime?	Yes	
		No	
2	If yes,		
2.1	Did you do it regularly, once a month	Yes	
		No	
2.2	Do you perform breast self-examination a few days after menses/specific date in a month (if your menses are irregular/menopause)?	Yes	
		No	
3	Have you ever had a mammography?	Yes	
